# District of Columbia

**Published:** 1899-05

**Authors:** J. P. Turner

**Affiliations:** Secretary


Secretary.

### VETERINARY ASSOCIATION OF THE DISTRICT OF COLUMBIA.

THE regular meeting was called to order by the Secretary in the absence of both President and Vice-President. Moved by Dr. Robinson, that Dr. Dawson preside over the meeting. The following were present: Drs. French, C. B. Robinson, J. Barton, Walmer, Reme, Turner, Gheen, Gelston, Dawson, and C. F. Hadfield. Visitors: Dr. Hill, of Rockville, and Mr. Flowers, student. Minutes of previous meeting were approved.

The Committee on Legislation reported that the Health Officer of the District of Columbia had the bill to regulate veterinary surgery under consideration, and that it would be presented at the next session of Congress, together with the dental bill.

The election of officers for the ensuing year resulted in the following: President, J. P. Turner; Vice-President, C. F. Dawson; Secretary and Treasurer, B. F. Gheen; Trustee (three years), E. Walmer.

Dr. Walmer addressed the Society on the use of mallein, and gave his experiences with it among the glandered horses of the Second United States Volunteer Cavalry at Jacksonville, Fla., last fall. The remarks of the doctor were chiefly confined to those cases which after injection with mallein gave unusual manifestions of body temperature, while the usual specific local reaction was present, and post-mortem examination proved the existence of glanders invariably. The talk was particularly interesting to the members, as it was based on the use of mallein upon five hundred horses. Dr. Barton moved a vote of thanks, which was unanimously given Dr. Walmer.

Dr. Dawson presented two very interesting specimens, and explained each. The first was the ossified pleura of a hog. It was convex and partially cavernous, shaping itself to the inner aspect of the ribs, and the cavernous portion contained a lobe of the lungs. The second specimen was a bracelet exostosis around the metacarpal bones. The bracelet was movable and was held in place by a wire hobble, which caused the growth, the calf having been picketed on a pasture. As the calf grew the wire had cut through the skin and caused periostitis by its pressure on the bones, resulting in this tumor. The exhibition of these specimens drew out many remarks, especially on the ossified pleura, it being considered almost as a pathological freak. Dr. Dawson was voted the thanks of the Association for his paper.

Dr. C B. Robinson cited a case of polypus of the trachea caused by penetration of a splinter of wood. The polypus had a pendulum motion during respiration, and interfered with the usefulness of the horse. Tracheotomy below the tumor, an incision along the jugular space, and opening of the trachea alongside of the tumor revealed the polypus. Operation was successful.

Papers for next meeting were furnished by Drs. Walmer, French, and Lockwood. Meeting then adjourned.

J. P. TURNER,
Secretary.

